# Effects of long-term application of traditional compost and bio-compost on soil microbial community structure and life-history strategies

**DOI:** 10.3389/fmicb.2026.1910948

**Published:** 2026-09-30

**Authors:** Lizhan Liu, Tao Yang, Xiaofang Wu, Yang Fan, Peipei Zhang, Jia Li, Wenbing Tan, Xiawei Peng

**Affiliations:** 1 Technical Centre for Soil, Agriculture and Rural Ecology and Environment, Ministry of Ecology and Environment, Beijing, China; 2 School of Biological Sciences and Technology, Beijing Forestry University, Beijing, China; 3 College of Resources and Environmental Sciences, China Agricultural University, Beijing, China; 4 State Environmental Protection Key Laboratory of Simulation and Control of Groundwater Pollution, Chinese Research Academy of Environmental Sciences, Beijing, China; 5 Institute of Tree Development and Genome Editing, Beijing Forestry University, Beijing, China; 6 Beijing Key Laboratory of Food Processing and Safety in Forestry, Beijing Forestry University, Beijing, China

**Keywords:** bio-compost, extracellular enzyme activities, life-history strategies, microbial community, traditional compost

## Abstract

**Introduction:**

Long-term compost application is an important strategy for improving soil fertility and regulating soil microbial communities, but the comparative effects of traditional compost and bio-compost on microbial life-history strategies remain unclear, which is a valuable framework for linking community shifts to soil carbon and nutrient cycling.

**Methods:**

In this study, a long-term (30-year) field experiment established in 1993 under a winter wheat–summer maize cropping system was used to evaluate the effects of high-rate bio-compost, high-rate traditional compost, low-rate bio-compost, low-rate traditional compost, and an unfertilized control on soil properties, enzyme activities, microbial community structure, and oligotrophic/copiotrophic groups.

**Results:**

Compost application significantly increased soil organic carbon, total nitrogen, total phosphorus, and soil moisture, with soil organic carbon content in the high-rate bio-compost treatment being 33.8% higher than that of the control. Soil oxidative enzyme activities, including catalase, lignin peroxidase, manganese peroxidase, and laccase, were also enhanced by compost addition. Bacterial diversity was highest under low-rate bio-compost, whereas fungal diversity and trophic strategies showed stronger responses to fertilization. Principal coordinate analysis and permutational multivariate analysis of variance indicated that both bacterial and fungal community compositions were significantly altered by 30 years of compost application. The bacterial oligotroph/copiotroph ratio remained relatively stable among treatments, while the fungal oligotroph/copiotroph ratio decreased significantly under low-rate bio-compost. Specifically, this treatment significantly decreased the relative abundance of the oligotrophic fungal phylum Basidiomycota while increasing the copiotrophic phylum Ascomycota, indicating a shift toward copiotrophic fungal groups. Co-occurrence network analysis showed that the high-rate bio-compost significantly enhanced microbial network complexity, increasing the number of nodes from 419 (control) to 753 and edges from 828 to 1841 in the bacterial network, suggesting enhanced microbial connectivity under high-rate bio-compost application. Correlation, Mantel, and redundancy analyses further identified soil total phosphorus and oxidative enzyme activities, especially manganese peroxidase, lignin peroxidase, and laccase, as key factors associated with microbial community variation and trophic group distribution.

**Conclusion:**

Overall, long-term bio-compost application improved soil fertility, stimulated oxidative enzyme activities, enhanced microbial network complexity, and regulated microbial life-history strategies, with different responses between bacterial and fungal communities.

## 1 Introduction

Long-term fertilization is a fundamental agricultural practice for sustaining soil fertility, crop productivity, and agroecosystem stability. Previous studies have shown that organic amendments can improve soil quality by supplying organic carbon, nitrogen, phosphorus, and other nutrients, while also enhancing soil physical and microbial activity (Lehmann et al., 2020; Islam et al., 2026). Compared with mineral fertilizers, organic amendments provide both readily available nutrients and relatively stable organic substrates, thereby influencing both soil chemical properties and biological processes (Diacono and Montemurro, 2010; Bebber and Richards, 2022). Among organic amendments, traditional compost has long been used as an important soil conditioner. In recent years, bio-compost, produced by adding microbial inoculants during composting, has attracted increasing attention because the introduced microorganisms can enhance composting efficiency and may accelerate organic matter decomposition and nutrient transformation more effectively than traditional compost (Bang et al., 2024; Salih et al., 2026). However, previous findings on bio-compost effects remain inconsistent, partly because its performance depends on application rate, soil type, crop system, and management history (Palansooriya et al., 2023; Gao et al., 2026). Therefore, direct comparisons between traditional compost and bio-compost under long-term field conditions are still needed.

Soil microorganisms are key drivers of soil nutrient cycling, organic matter turnover, and ecosystem functioning. Microbial communities respond sensitively to compost-induced changes and are closely linked to carbon and nutrient turnover (Guo et al., 2024). Thus, understanding these responses is essential for evaluating the long-term ecological effects of compost application. Numerous studies have demonstrated that long-term organic fertilization can increase microbial biomass, alter microbial diversity, and shift bacterial and fungal community composition by changing substrate availability, soil pH, moisture, and nutrient stoichiometry (Qaswar et al., 2021; Cui H. et al., 2023). Bacteria are generally sensitive to changes in labile carbon and nutrient availability, whereas fungi are more closely involved in the decomposition of complex organic materials and may respond differently to compost-induced changes in organic matter quality (Liu et al., 2022). In addition, extracellular enzymes provide an important functional link between microbial communities and soil nutrient transformation. For example, catalase is associated with oxidative stress regulation, while lignin peroxidase, manganese peroxidase, and laccase participate in the oxidative degradation of complex organic substrates such as lignin-derived compounds (Galazka et al., 2025). Previous research has reported that compost application can enhance soil enzyme activities by increasing substrate supply and improving nutrient availability (Liu S. B. et al., 2020; Lucchetta et al., 2023). However, how changes in enzyme activities are linked with bacterial and fungal community shifts under different compost types and rates remains insufficiently clarified.

Microbial life-history strategies provide a useful framework for interpreting microbial responses to resource availability. Copiotrophic microorganisms generally grow rapidly under nutrient-rich conditions and are favored by high substrate availability, whereas oligotrophic microorganisms are better adapted to nutrient-poor or relatively stable environments (Malik et al., 2020). The r/K life-history strategy framework provides an ecological perspective for assessing how changes in soil resource conditions induced by compost application favor either r-strategists, which grow rapidly and are sensitive to resource fluctuations, or K-strategists, which are characterized by higher resource-use efficiency and slower growth rates. This framework has been widely used to explain microbial community shifts following fertilization, organic amendment, land-use change, and soil restoration (He et al., 2025). Previous studies have suggested that nutrient enrichment often increases the relative abundance of copiotrophic taxa, such as Proteobacteria, Bacteroidota, and Firmicutes, while reducing or restructuring oligotrophic groups such as Acidobacteriota and Chloroflexi (Lori et al., 2023). In fungal communities, Ascomycota is frequently associated with copiotrophic strategies because many members respond rapidly to easily available substrates, whereas Basidiomycota is often considered more oligotrophic due to its ability to degrade complex organic compounds under relatively nutrient-limited conditions (Yao et al., 2017). Nevertheless, microbial trophic classifications are not always consistent across ecosystems, and long-term compost application may simultaneously increase nutrient availability and complex organic substrates, leading to more complicated responses of oligotrophic and copiotrophic groups.

In addition to diversity and community composition, microbial co-occurrence networks provide insights into potential associations among microbial taxa. Previous studies have increasingly used network analysis to evaluate how fertilization, organic amendments, and environmental changes influence microbial interaction patterns (Luo et al., 2022; Khan et al., 2025). Network complexity, usually reflected by the number of nodes, edges, and the balance between positive and negative associations, can indicate the extent of microbial connections under different soil management practices (Wang et al., 2024). Positive correlations in co-occurrence networks are often interpreted as potential co-occurrence, shared environmental preferences, or cooperative relationships, whereas negative correlations may reflect potential competition, niche differentiation, or exclusion effects (Xiong et al., 2022). Long-term organic amendments may increase network complexity by providing diverse organic substrates and creating more heterogeneous soil microhabitats, but responses may differ between bacteria and fungi and may also depend on compost type and application rate (Liu Z. et al., 2020; Kang et al., 2021). Despite these advances, few studies have integrated bacterial and fungal diversity, trophic strategies, co-occurrence networks, extracellular enzyme activities, and soil environmental drivers within the same long-term compost experiment.

In this study, we used a long-term field experiment established in 1993 under a winter wheat–summer maize double-cropping system to investigate the effects of traditional compost and bio-compost at two application rates. We measured soil physicochemical properties, extracellular enzyme activities, bacterial and fungal alpha diversity, community composition, oligotrophic and copiotrophic microbial groups, and microbial co-occurrence networks. The objectives were to: (1) determine how long-term traditional compost and bio-compost applications affect soil nutrient status and extracellular enzyme activities; (2) evaluate the responses of bacterial and fungal diversity, community composition, and co-occurrence networks to different compost treatments; and (3) identify the key soil environmental factors and enzyme activities associated with microbial trophic groups. We hypothesized that long-term compost application, particularly high-rate bio-compost, would improve soil nutrient availability and enzyme activities, reshape bacterial and fungal community structures, enhance microbial network complexity, and alter the balance between oligotrophic and copiotrophic microbial groups.

## 2 Materials and methods

### 2.1 Site description and experimental design

This study was conducted at the Quzhou Experimental Station of China Agricultural University, located in Quzhou County, Handan City, Hebei Province, China (Supplementary Figure S1). The station is situated in a typical temperate monsoon climate zone, characterized by cold and dry winters and hot, rainy summers, with precipitation mainly concentrated in summer. The mean annual precipitation is approximately 550 mm, and the mean annual temperature is 13.1 °C. The soil used in this study was classified as cinnamon soil.

The experimental field follows a winter wheat–summer maize double-cropping system, and the long-term (30-year) fertilization experiment was initiated in 1993. All fertilization treatments have been continuously maintained with the same application rates and frequencies since the initiation of the experiment. Five treatments were established: a high-rate bio-compost treatment (B15), a high-rate traditional compost treatment (T15), a low-rate bio-compost treatment (B7.5), a low-rate traditional compost treatment (T7.5), and an unfertilized control (CK), with four replicates for each treatment. All fertilization treatments were applied twice annually, before maize sowing in June and before wheat sowing in October. Crop management practices, including pest and weed control, were applied uniformly across all treatments. The field was managed under conventional tillage, which involved deep plowing followed by harrowing and leveling prior to wheat sowing, and rotary tillage prior to maize sowing. No inorganic fertilizers or other soil amendments were applied throughout the experimental period. The traditional compost was produced through aerobic composting of maize straw, chicken manure, brown sugar, soybean meal, and cottonseed meal in a ratio of 60:30:1:4:5 (w/w) until fully mature. These feedstocks were selected for their local availability and complementary nutrient composition to achieve a balanced C/N ratio and support microbial activity during composting. The bio-compost was produced by adding 200 mL of microbial inoculant (containing 2 × 10^8^ CFU/mL beneficial microorganisms including *Aspergillus niger*, *Saccharomyces cerevisiae*, *Lactobacillus plantarum*, *Lactobacillus acidophilus*, *Bacillus megaterium*, *Streptomyces albogriseolus*, *Bacillus subtilis*, Beijing VOTO Biotech Co. Ltd., Beijing, China) per 100 kg of conventional compost raw material prior to fermentation. The basic physicochemical properties of the two compost types are presented in Supplementary Table S1. Bio-compost was characterized by substantially higher organic matter and available phosphorus contents compared to traditional compost, while total nitrogen, available potassium, pH, and moisture content were comparable between the two compost types. The analytical methods are described in Section 2.3. Detailed fertilization conditions and application rates for each treatment are shown in Supplementary Table S2. The five treatments included high-rate (15 t/ha) and low-rate (7.5 t/ha) applications of both bio-compost and traditional compost, alongside an unfertilized control (CK).

### 2.2 Soil sampling

Soil samples were collected in October 2024, during the seedling stage of winter wheat—a period when crop nutrient demand is low and interference from mature root systems is minimal, allowing the early responses of soil properties and microbial communities to compost application to be captured. Soil samples were collected from each plot using a random sampling method. Five sampling points were selected within each plot, and soil samples were collected from the 0–20 cm plow layer using a manual soil auger. Before sampling, surface litter and other debris were removed. Soil collected from the five points within each plot was then thoroughly mixed to form one composite sample representing that plot. After transport to the laboratory, visible plant residues, plastic film fragments, and other animal or plant debris were removed, and the samples were passed through a 2 mm sieve. Air-dried subsamples were used for the determination of soil physicochemical properties, fresh subsamples stored at 4 °C were used for soil enzyme activity assays, and subsamples stored at −20 °C were used for microbial DNA extraction.

### 2.3 Determination of physicochemical properties and enzyme activities

Soil organic carbon (SOC) was determined using the potassium dichromate oxidation method with external heating, total nitrogen (TN) was measured by the Kjeldahl method, and total phosphorus (TP) was determined using the acid digestion–molybdenum antimony colorimetric method. Based on the measured values of SOC, TN, and TP, the stoichiometric ratios of SOC: TN, TC: TP, and TN: TP were further calculated to characterize soil nutrient stoichiometry. Soil pH was measured potentiometrically in a soil–water suspension at a ratio of 1:2.5 (w/v) using a pH meter. Soil moisture content was determined by the oven-drying method, in which fresh soil samples were dried at 105 °C to constant weight.

Soil enzyme activities were determined using substrate-based colorimetric assays, including catalase (Cat), lignin peroxidase (LiP), manganese peroxidase (MnP), and laccase (Lac). All enzyme activity assays were performed using fresh soil samples. Under appropriate buffer conditions and constant-temperature incubation, specific substrates were added to initiate enzymatic reactions, and the absorbance changes in the reaction system were subsequently measured using a spectrophotometer. Enzyme activities were then calculated based on standard curves or the corresponding conversion formulas. Specifically, CAT activity was characterized by its ability to decompose hydrogen peroxide (Sinha, 1972); LiP activity was determined based on the changes in products formed during the oxidation of lignin-like substrates (Tien and Kirk, 1988); MnP activity was assessed by measuring the formation of reaction products associated with Mn^2+^ oxidation (Wariishi et al., 1992); and Lac activity was determined by monitoring absorbance changes during the oxidation of phenolic substrates (Bourbonnais and Paice, 1990). Blank controls and replicate samples were included in all enzyme activity assays. Enzyme activities were expressed as the amount of substrate transformed or product generated per unit soil mass per unit time, in order to reflect the characteristics and differences of soil oxidative enzyme activities under different fertilization treatments.

Organic matter (OM) in the compost materials was determined by the loss-on-ignition method. TN was determined by the Kjeldahl method. Available phosphorus (AP) was extracted using the Olsen method and determined by molybdenum blue colorimetry. Available potassium (AK) was extracted with ammonium acetate and measured by flame photometry. Compost pH was measured potentiometrically in a 1:5 (w/v) compost-to-water suspension, and moisture content was determined by oven-drying at 105 °C to constant weight.

### 2.4 Soil microbial community analyses

Soil DNA was extracted from approximately 0.20 g of frozen soil (−80 °C) using the PowerSoil DNA Isolation Kit (MoBio Laboratories, Carlsbad, CA, United States). For bacteria, the V3–V4 region of the 16S rRNA gene was amplified using the primer pair 338F (5′-ACTCCTACGGGAGGCAGCAG-3′) and 806R (5′-GGACTACHVGGGTWTCTAAT-3′), and the amplicons were sequenced on the Illumina platform (Illumina, San Diego, CA, United States). For fungi, the internal transcribed spacer 1 (ITS1) region was amplified using the primer pair ITS1F (5′-CTTGGTCATTTAGAGGAAGTAA-3′) and ITS2R (5′-GCTGCGTTCTTCATCGATGC-3′). Raw sequences longer than 300 bp with an average quality score above 30 were processed using the Quantitative Insights Into Microbial Ecology (QIIME) pipeline. Amplicon sequence variants (ASVs) were clustered at 99% sequence similarity using UPARSE. Taxonomic assignment was performed against a subset of the SILVA 128 database for bacteria and the UNITE database for fungi. The raw reads generated in this study were deposited in the NCBI Sequence Read Archive (SRA) under accession number PRJNA1510276.

The oligotrophic and copiotrophic classification is grounded in the r/K selection framework, which distinguishes microorganisms by their growth and resource utilization strategies (Fierer et al., 2007; Couso et al., 2023). Based on previous studies, Alphaproteobacteria, Gammaproteobacteria, Bacteroidota, and Firmicutes were classified as copiotrophic bacteria, whereas Actinobacteriota, Verrucomicrobiota, Acidobacteriota, Gemmatimonadota, Chloroflexi, and Planctomycetota were classified as oligotrophic bacteria. For fungi, Ascomycota was considered a copiotrophic group, while Basidiomycota was regarded as an oligotrophic group. The relative abundances of dominant bacterial phyla/classes and fungal phyla were visualized using bar plots to illustrate shifts in microbial community composition.

### 2.5 Statistical analysis

Before analysis, normal distribution and homogeneity were tested for all data. One-way analysis of variance (ANOVA) was used to evaluate the effects of different fertilization treatments on soil physicochemical properties, enzyme activities, microbial alpha diversity indices, and the relative abundances of oligotrophic and copiotrophic microbial groups. When significant differences were detected, Tukey’s honestly significant difference (HSD) test was performed for multiple comparisons among treatments at *p* < 0.05. Principal coordinate analysis (PCoA) based on Bray–Curtis dissimilarity was performed to visualize differences in bacterial and fungal community compositions among treatments, and permutational multivariate analysis of variance (PERMANOVA) was used to test the significance of treatment effects on microbial community structure. Co-occurrence networks were constructed separately for bacterial and fungal communities under each treatment based on significant correlations among microbial taxa. For each treatment, ASVs with a prevalence of less than 20% across samples were removed, and only ASVs with a relative abundance ≥ 0.01% in at least one sample were retained. To mitigate the statistical limitations associated with network construction from a modest number of field replicates (*n* = 4), which is a common constraint in soil microbial studies (Faust, 2021), we adopted a conservative and stringent filtering strategy. Spearman’s rank correlation coefficients were calculated between all pairs of retained ASVs for each treatment separately. Only statistically significant correlations (*p* < 0.05) with a correlation coefficient |ρ| ≥ 0.7 were considered for network construction. Network parameters, including the number of nodes, number of edges, and proportions of positive and negative edges, were used to compare microbial network complexity among treatments. The networks were visualized using the ‘ggraph’ package, and topological parameters were calculated using the ‘igraph’ package in R (v4.2.3) (Csardi and Nepusz, 2006). Pearson correlation analysis was conducted to examine the relationships between microbial alpha diversity, soil physicochemical properties, and enzyme activities. Mantel tests were used to assess the associations between bacterial and fungal community compositions and environmental variables. Redundancy analysis (RDA) was further performed to explore the relationships between microbial trophic groups and soil environmental factors or enzyme activities. The significance of the RDA model, ordination axes, and individual environmental variables was tested using permutation tests. The independent explanatory power of each environmental factor was calculated using single-factor RDA. All statistical analyses and visualizations were performed in R (R version 4.2.3).

## 3 Results

### 3.1 Soil physicochemical properties and extracellular enzyme activities

Different fertilization treatments significantly affected soil physicochemical properties (Table 1). Compared with CK, compost application generally increased SOC, TN, TP, and soil moisture, with B15 exhibiting the highest values and T15 ranking second, both exceeding the control. SOC: TN and TC: TP did not differ significantly among treatments, whereas TN: TP was significantly higher in B15 and T15 than in CK and T7.5. Soil pH was higher in CK and B15 but lower in the other amended soils. Soil moisture was significantly higher in B15 and T15 than in the other treatments (*p* < 0.05). Overall, both traditional compost and bio-compost improved soil nutrient status, with stronger effects at the higher application rate, particularly under B15.

**Table 1:** Effects of different treatments on soil physicochemical properties.

Treatment	CK	B15	T15	B7.5	T7.5
SOC (g/kg)	8.81 ± 1.07c	11.79 ± 0.77a	11.14 ± 1.62ab	10.02 ± 0.21abc	9.42 ± 0.47bc
TN (g/kg)	1.06 ± 0.04b	1.57 ± 0.13a	1.52 ± 0.17a	1.26 ± 0.04b	1.17 ± 0.13b
TP (g/kg)	0.49 ± 0.02d	0.63 ± 0.01a	0.61 ± 0.01ab	0.58 ± 0.01c	0.59 ± 0.01bc
SOC: TN	8.32 ± 0.69a	7.58 ± 1.15a	7.28 ± 0.26a	7.99 ± 0.45a	8.10 ± 0.51a
SOC: TP	17.83 ± 1.62a	18.70 ± 1.37a	18.15 ± 2.30a	17.24 ± 0.79a	15.99 ± 0.48a
TN: TP	2.14 ± 0.02b	2.49 ± 0.18a	2.49 ± 0.23a	2.16 ± 0.02ab	1.98 ± 0.18b
pH	7.59 ± 0.03a	7.71 ± 0.19a	7.28 ± 0.02b	7.19 ± 0.09b	7.30 ± 0.15b
Moisture (%)	21.65 ± 0.24b	23.30 ± 1.08a	23.48 ± 0.20a	21.37 ± 0.09b	22.08 ± 0.27b

Different lowercase letters indicate significant differences among treatments based on Tukey’s HSD test at *p* < 0.05.

Soil extracellular enzyme activities varied significantly among fertilization treatments (Figure 1a). Overall, compost application enhanced Cat, LiP, MnP, and Lac compared with CK. In general, the high-rate compost treatments (B15 and T15) showed greater enzyme activities than the low-rate treatments (B7.5 and T7.5) (*p* < 0.05). Among the treatments, B15 exhibited the strongest stimulatory effect on Cat and MnP activities, while LiP and Lac activities were also generally elevated under compost addition relative to CK. These results indicate that long-term compost application, particularly bio-compost at the higher rate, promoted soil oxidative enzyme activities.

**Figure 1:**
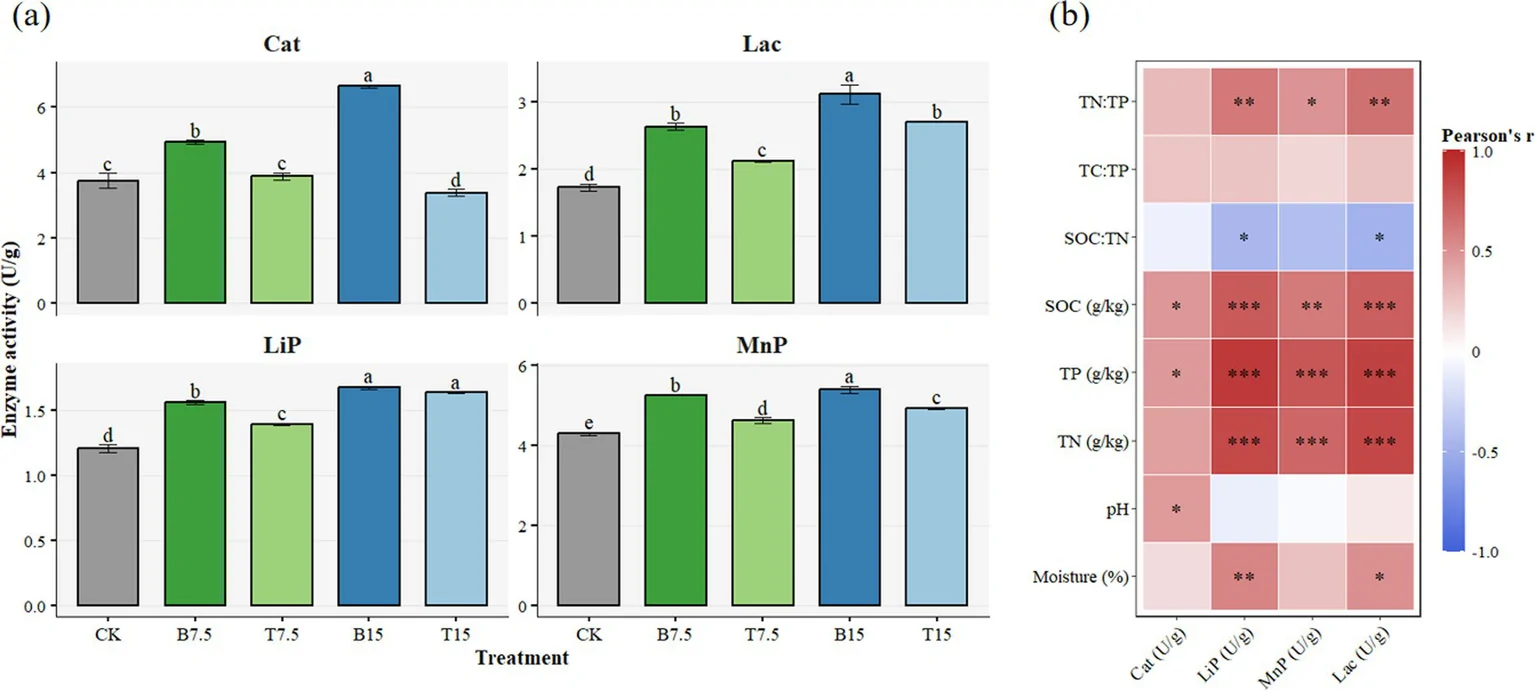
Circular visualization of **(a)** soil extracellular enzyme activities under different treatments and **(b)** Pearson correlations between enzyme activities and soil physicochemical properties. Cat, catalase; LiP, lignin peroxidase; MnP, manganese peroxidase; Lac, laccase. Different lowercase letters indicate significant differences among treatments based on Tukey’s HSD test at *p* < 0.05. The color scale represents the strength and direction of the correlations, and asterisk denote significance levels (*p < 0.05, **p < 0.01, ***p < 0.001).

Correlation analysis further showed that soil enzyme activities were closely associated with soil physicochemical properties (Figure 1b). Cat, LiP, MnP, and Lac were generally positively correlated with SOC, TN, TP, TN: TP, TC: TP, soil moisture, and pH, whereas negative correlations were observed with SOC: TN (*p <* 0.05). Among these relationships, SOC, TN, and TP showed relatively strong positive correlations with oxidative enzyme activities, while SOC: TN exhibited negative associations, especially with LiP and MnP. These results suggest that improvements in soil nutrient status and moisture under compost application were accompanied by enhanced oxidative enzyme activities.

### 3.2 Soil bacterial and fungal alpha diversity and community structure

Different fertilization treatments significantly affected the alpha diversity of soil bacterial and fungal communities (Figure 2). For bacteria, Shannon and Chao1 indices were highest under B7.5 and lowest under T15 (*p* < 0.05), with B15 and T7.5 showing intermediate values (Figures 2a,b). For fungi, Shannon was significantly lower in T7.5 than in CK and B15 (*p* < 0.05), while Chao1 did not differ significantly among treatments (Figures 2d,e). Principal coordinates analysis further revealed clear shifts in both bacterial and fungal community composition under different fertilization treatments (Figures 2c,f). For bacteria, the first two axes explained 21.9 and 9.0% of the total variation, respectively, and PERMANOVA confirmed that fertilization treatments significantly affected bacterial community structure (*R*^2^ = 0.39, *p* < 0.001). For fungi, PCoA1 and PCoA2 explained 31.8 and 21.2% of the variation, respectively, and fungal community structure also differed significantly among treatments (PERMANOVA, *R*^2^ = 0.75, *p* < 0.001). Overall, these results indicate that long-term compost application altered both the alpha diversity and community composition of soil bacteria and fungi, although the response patterns differed between bacterial and fungal communities.

**Figure 2:**
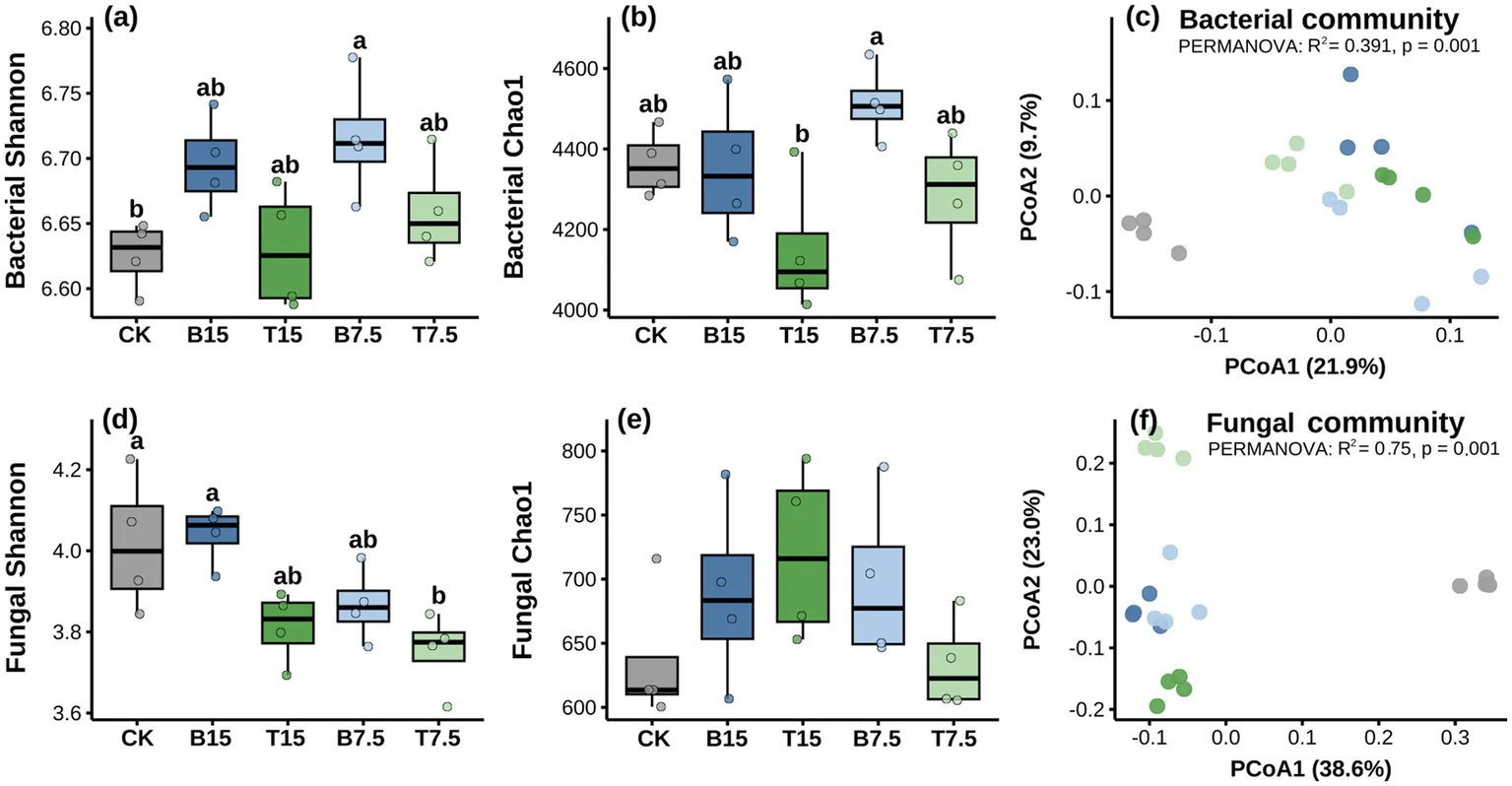
Effects of different treatments on soil bacterial and fungal alpha diversity and community structure. **(a)** Bacterial Shannon index; **(b)** bacterial Chao1 richness; **(c)** principal coordinate analysis (PCoA) of bacterial community composition based on Bray–Curtis dissimilarity; **(d)** fungal Shannon index; **(e)** fungal Chao1 richness; **(f)** PCoA of fungal community composition based on Bray–Curtis dissimilarity. Different lowercase letters denote significant differences among treatments according to Tukey’s HSD test at *p* < 0.05.

The relative abundances of oligotrophic and copiotrophic microbial groups differed among fertilization treatments (Figure 3). Among the oligotrophic bacterial phyla (Figure 3a), Actinobacteriota dominated and remained stable across treatments. Chloroflexi and Planctomycetota were highest in CK, while Acidobacteria was more abundant in B15 and B7.5 than in CK. Gemmatimonadota showed no significant difference among treatments. For copiotrophic bacterial groups (Figure 3b), the relative abundance of Firmicutes was significantly higher in B15 than in B7.5. Alphaproteobacteria was significantly more abundant in CK than in B15. The relative abundances of Gammaproteobacteria and Bacteroidota were significantly higher in B7.5 than in CK. However, the ratio of oligotrophic to copiotrophic bacterial groups showed no significant difference among fertilization treatments (Figure 3c).

**Figure 3:**
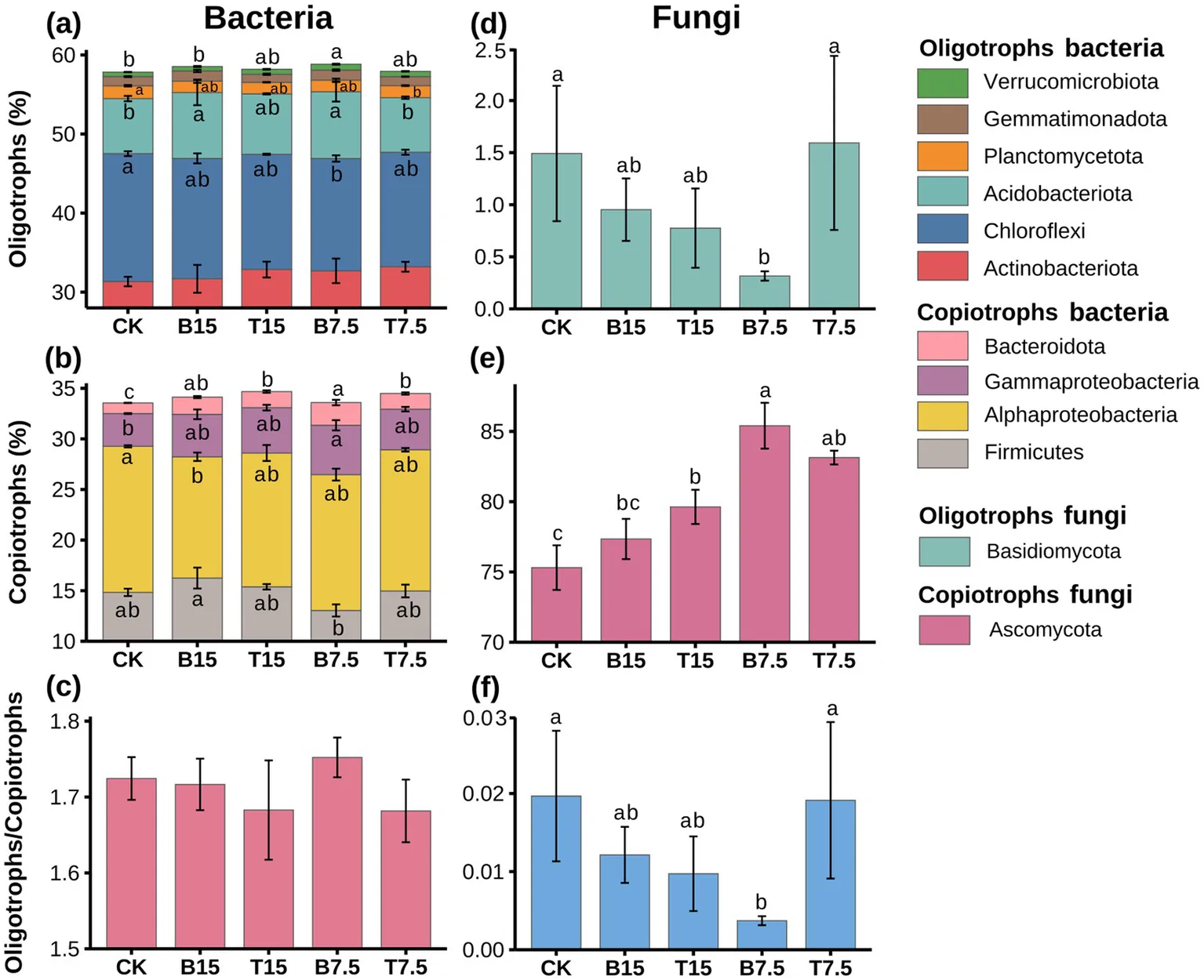
Relative abundances of oligotrophic and copiotrophic bacteria and fungi, and the ratio of oligotrophs to copiotrophs under different treatments. **(a)** Relative abundance of oligotrophic bacterial phyla. **(b)** Relative abundance of copiotrophic bacterial groups. **(c)** Bacterial oligotroph/copiotroph ratio. **(d)** Relative abundance of the oligotrophic fungal phylum Basidiomycota. **(e)** Relative abundance of the copiotrophic fungal phylum Ascomycota. **(f)** Fungal oligotroph/copiotroph ratio. Significant differences at the phylum (or subphylum) level among different treatments are denoted by lowercase letters (*p* < 0.05).

In fungal communities, the oligotrophic group, represented by Basidiomycota, responded significantly to fertilization. Its relative abundance was significantly lower in B7.5 than in CK and T7.5 (Figure 3d). In contrast, the copiotrophic fungal group, represented by Ascomycota, showed the opposite trend. Ascomycota was significantly more abundant in B7.5 than in CK, T15 and B15 (Figure 3e). Consistently, the fungal oligotroph/copiotroph ratio was significantly lower in B7.5 (Figure 3f).

### 3.3 Co-occurrence networks of bacterial and fungal communities

Co-occurrence networks revealed clear differences in microbial network complexity among fertilization treatments (Figure 4). B15 exhibited the highest complexity (753 nodes, 1841 edges), followed by B7.5 (586 nodes, 1,116 edges), while CK (419 nodes, 828 edges), T15 (475 nodes, 739 edges), and T7.5 (448 nodes, 721 edges) were simpler. Positive correlations dominated the bacterial networks across all treatments, accounting for 91.55–97.09% of total edges, whereas negative correlations accounted for only 2.91–8.45%. The highest proportion of negative edges was observed in CK, suggesting relatively stronger potential competitive or antagonistic interactions under the control treatment. For the fungal networks, B15 was the most complex (386 nodes, 489 edges), whereas CK was the simplest (87 nodes, 87 edges). T15, B7.5, and T7.5 showed intermediate network sizes. Similar to the bacterial networks, fungal networks were also dominated by positive correlations, which accounted for 95.03–98.56% of total edges. Negative correlations were relatively low, ranging from 1.44 to 4.97%, with the highest proportion observed under T15 and the lowest under B15.

**Figure 4:**
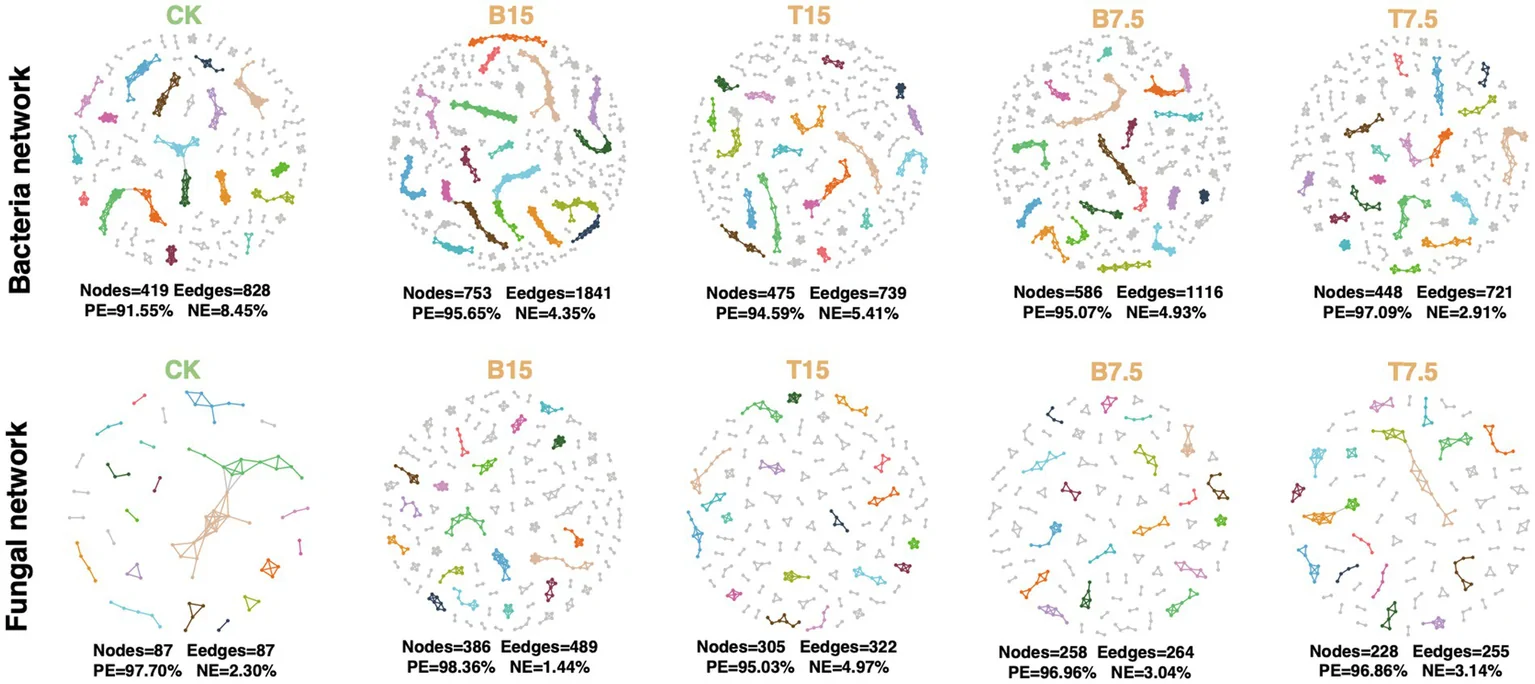
Co-occurrence networks of bacterial and fungal communities under different fertilization treatments. In the network, nodes represent the microbial ASVs, edges represent the significant correlations between ASVs. PE and NE represent the proportion of positive edges and negative edges, respectively.

### 3.4 Relationships among soil microbial community and edaphic properties

Pearson correlation analysis showed significant correlations between microbial alpha diversity and soil environmental factors or enzyme activities (Figure 5a). Bacterial Shannon diversity was positively correlated with Cat and MnP activities (*p* < 0.01), whereas bacterial Chao1 richness was negatively correlated with moisture (*p* < 0.01) and positively correlated with Cat activity (*p* < 0.05). Fungal Shannon diversity was positively correlated with SOC: TP and pH (*p* < 0.05). Fungal Chao1 richness was positively correlated with SOC, TP, LiP, Lac, TN, TN: TP, with the strongest correlation observed for TN: TP (*p* < 0.05).

**Figure 5:**
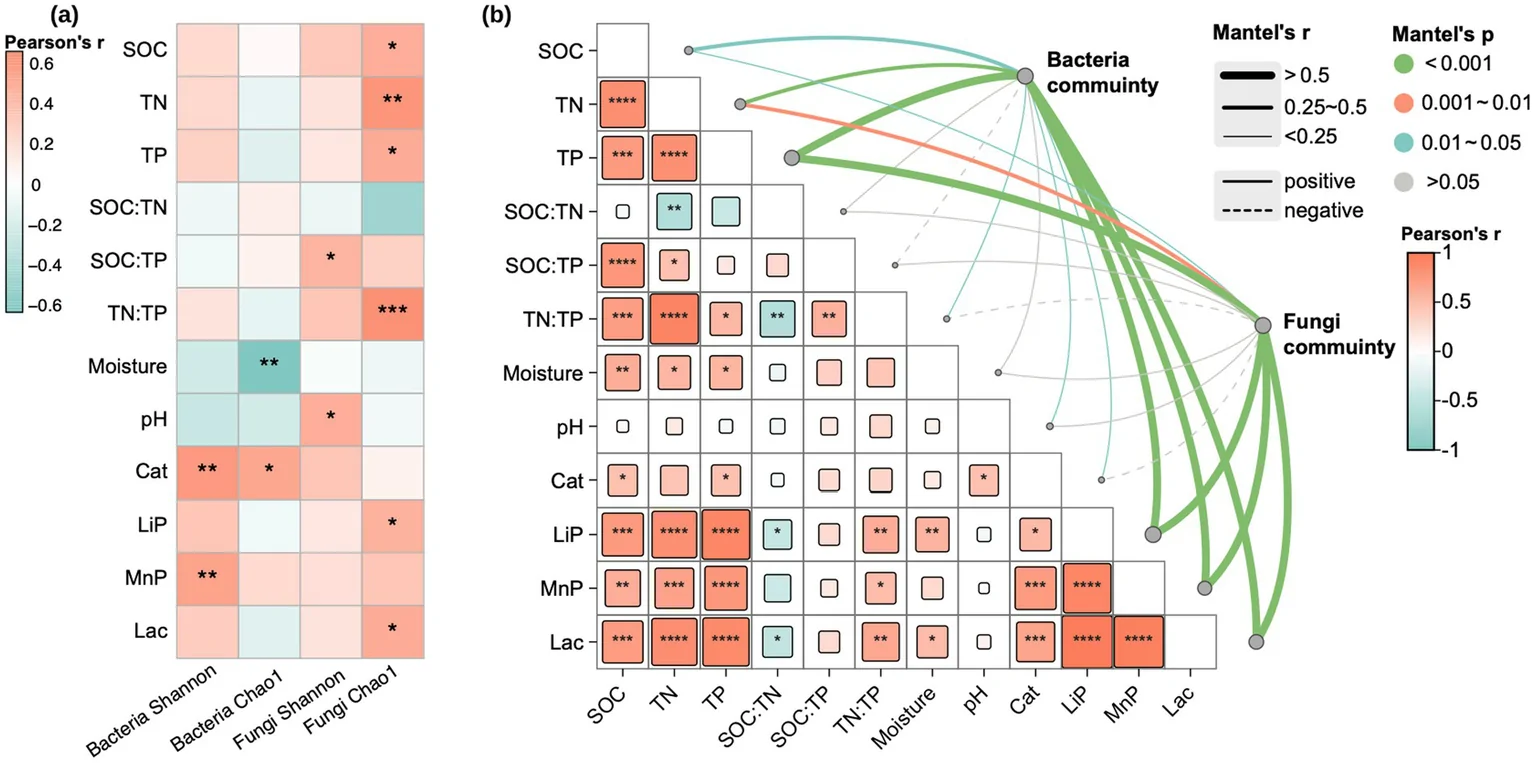
Correlation analysis between soil environmental factors and microbial community diversity and composition, including **(a)** Pearson correlations between microbial alpha diversity indices and soil environmental factors or enzyme activities, and **(b)** mantel tests linking these variables to bacterial and fungal community composition.

Mantel test results showed that both bacterial and fungal community compositions were significantly associated with soil environmental factors and enzyme activities (Figure 5b). The bacterial community was significantly correlated with TP, LiP, MnP, and Lac (*p* < 0.001), and also showed significant associations with SOC: TN, TN: TP, and pH (*p* < 0.05). Among these factors, TP showed the strongest association with bacterial community composition. The fungal community was significantly correlated with TP, LiP, MnP, and Lac (*p* < 0.001), indicating strong associations between fungal community composition and soil phosphorus status as well as extracellular enzyme activities. In contrast, SOC, TN, moisture, and pH showed weak or non-significant relationships with fungal community composition (*p* > 0.05).

Redundancy analysis (RDA) results showed that soil environmental factors and enzyme activities explained a large proportion of the variation in microbial trophic groups (Figure 6). The first two RDA axes explained 75.34% of the total constrained variation, with RDA1 and RDA2 accounting for 52.70 and 22.64%, respectively (Figure 6a). In the RDA ordination, MnP, LiP, Lac, and TP were mainly positively associated with RDA1, whereas pH, SOC: TN, and SOC: TP were negatively associated with RDA1. Moisture was mainly positively associated with RDA2, while TN and TP were negatively associated with RDA2. Among microbial taxa, Acidobacteriota, Ascomycota, and Bacteroidota clustered on the positive side of RDA1 with Cat, LiP, Lac, MnP, and TP. In contrast, Basidiomycota, Alphaproteobacteria, and Chloroflexi were on the negative side with pH, SOC: TN, and SOC: TP. The independent explanation analysis showed that MnP had the highest explanatory power for microbial trophic group variation, explaining 15.5% of the variation, followed by LiP and Lac, which explained 12.8 and 12.2%, respectively (Figure 6b, *P* < 0.05), whereas the remaining variables explained 3.8–9.2% of the variation (Figure 6b, *P* > 0.05).

**Figure 6:**
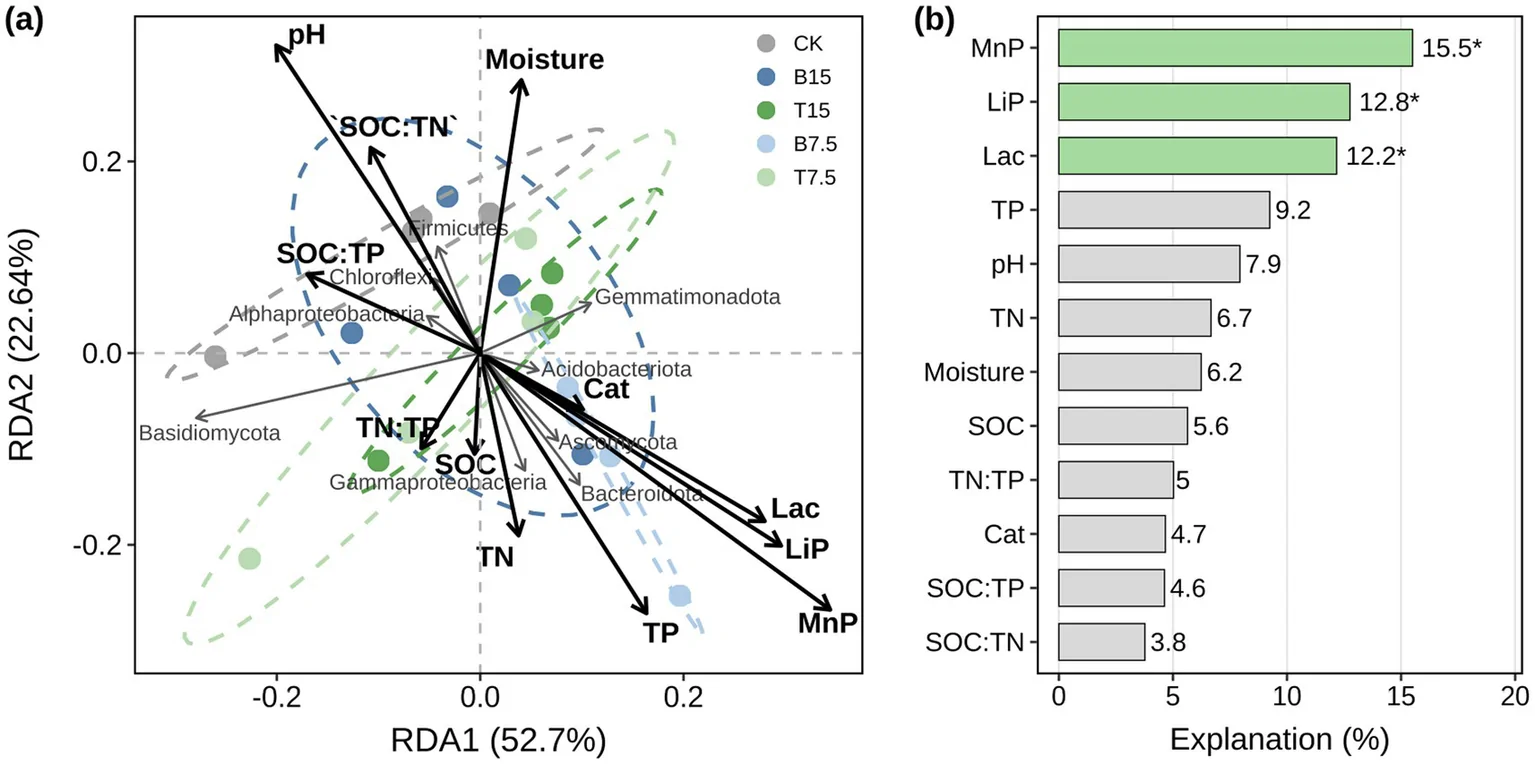
Redundancy analysis (RDA) of microbial trophic groups in relation to soil environmental factors and enzyme activities, including **(a)** the RDA ordination showing the associations among microbial trophic groups, fertilization treatments, environmental variables, and enzyme activities, and **(b)** the independent explanatory power of each environmental factor or enzyme activity based on single-factor RDA.

## 4 Discussion

Long-term compost application significantly improved soil nutrient status, as shown by the increases in SOC, TN, TP, and soil moisture compared with the unfertilized control. This result is consistent with previous studies reporting that organic amendments enhance soil fertility through direct inputs of organic matter and nutrients, while also improving soil physical properties such as water retention and aggregate stability (Qiang et al., 2024; Munna and Lal, 2025). In this study, the high-rate compost treatments, especially B15, showed the strongest increases in SOC, TN, and TP, indicating that the magnitude of soil nutrient improvement was closely related to compost input level and compost type. The distinct nutrient profiles of the compost materials clearly underpinned the divergent effects of the treatments on soil fertility. Bio-compost was characterized by substantially higher OM and AP contents compared to traditional compost. This nutrient-rich profile directly contributed to the superior SOC accumulation and the significantly highest soil TP levels observed under the B15 treatment. Notably, these findings underscore the critical role of high-rate bio-compost applications in shifting the soil system toward a high-carbon and high-phosphorus equilibrium, which is favorable for sustaining the long-term productivity of intensive wheat-maize cropping systems (Chen et al., 2024). The higher SOC and TN under B15 and T15 suggest that long-term organic material addition promoted carbon and nitrogen accumulation in the plow layer. Meanwhile, the increase in TP under all compost treatments reflects the direct phosphorus input from compost and its long-term accumulation in agricultural soils (Bhogal et al., 2018; Hsiao et al., 2025). Soil pH also differed among treatments, suggesting that compost type and application rate may alter soil acid–base conditions through changes in organic acid production and microbial decomposition processes (López et al., 2021). Overall, these findings confirm that long-term compost application can improve soil nutrient supply, with high-rate bio-compost showing particularly strong effects on soil fertility.

The enhancement of soil extracellular enzyme activities further indicates that compost application stimulated soil biological functioning. Catalase, lignin peroxidase, manganese peroxidase, and laccase activities were generally higher in compost-amended soils than in CK, with the high-rate treatments showing stronger effects than the low-rate treatments. This pattern is likely related to the increased availability of organic substrates and nutrients, which can promote microbial growth and enzyme production (Galazka et al., 2025). Oxidative enzymes such as LiP, MnP, and Lac are involved in the degradation of lignin-like and other complex organic compounds, and their stimulation under compost application suggests an enhanced capacity for organic matter transformation (Zeng et al., 2018). The positive correlations between enzyme activities and SOC, TN, TP, moisture, and TN: TP further support the close linkage between improved soil nutrient conditions and enzyme-mediated processes. Among treatments, B15 exhibited the strongest promotion of Cat and MnP activities, suggesting that bio-compost at a higher rate may provide more favorable conditions for oxidative microbial metabolism. This enhancement is likely driven by the nutrient-rich bio-compost matrix, which provided greater OM and readily available AP inputs, along with the functional microbial additives. The high nutrient density and specific microbial consortia in bio-compost may stimulate soil enzyme production by providing both abundant substrates and effective decomposer communities (Zhang et al., 2023). It should be noted, however, that while microbial inoculants can influence decomposition processes, their long-term survival and direct contribution require further verification (Zhang et al., 2021).

Long-term compost application also altered microbial alpha diversity and community composition, but bacterial and fungal communities responded differently. Bacterial Shannon diversity and Chao1 richness were highest in B7.5, suggesting that moderate bio-compost application may create favorable conditions for bacterial diversity. This may reflect a balance between increased resource availability and reduced environmental filtering: low-rate bio-compost can supply organic substrates and nutrients without causing excessive nutrient accumulation or strong shifts in soil chemical conditions. Previous studies have shown that moderate organic amendment can increase bacterial diversity by broadening niche availability, whereas excessive fertilization may reduce diversity through stronger selection for nutrient-responsive taxa (Mei et al., 2021). In contrast, fungal Shannon diversity was lower in T7.5 than in CK and B15, while fungal Chao1 richness did not differ significantly among treatments. This indicates that fertilization mainly changed fungal dominance structure rather than total richness. The PCoA and PERMANOVA results further confirmed that fertilization significantly shifted both bacterial and fungal community structures, with fungal communities showing a stronger treatment effect. This may be because fungal communities, as the primary decomposers of complex organic polymers, are more sensitive to the modifications in organic matter quality, pH, and oxidative decomposition processes (Guo et al., 2023). Consequently, the stronger response of fungal community structure suggests that long-term organic management strategies primarily alter the functional trajectories of decomposition pathways within the soil food web. Collectively, compost application reshaped the microbial community, but the direction and magnitude of the response depended on microbial group and fertilization regime.

The classification of microbial taxa into oligotrophic and copiotrophic groups provides a useful perspective for understanding how compost application affected microbial life-history strategies. In bacterial communities, oligotrophic taxa remained more abundant than copiotrophic taxa across all treatments, and the oligotroph/copiotroph ratio did not differ significantly among treatments. This suggests that long-term compost application changed the composition of specific bacterial groups but did not fundamentally shift the overall bacterial trophic balance. Actinobacteriota was the dominant oligotrophic group and remained stable among treatments, reflecting its broad adaptability and potential ability to utilize diverse carbon sources under different nutrient conditions (Zhao et al., 2024). In contrast, Chloroflexi and Planctomycetota were more abundant in CK, whereas Acidobacteriota increased under B15 and B7.5, indicating that different oligotrophic groups responded differently to compost-induced environmental changes. For copiotrophic bacteria, B7.5 increased Gammaproteobacteria and Bacteroidota, while B15 favored Firmicutes relative to B7.5. These taxa are often associated with rapid responses to available organic substrates and nutrient enrichment (Shu et al., 2023). However, the stable bacterial oligotroph/copiotroph ratio suggests that compost-amended soils may contain both labile and recalcitrant organic substrates, supporting the coexistence of contrasting bacterial strategies. Fungal trophic strategies showed a clearer response to compost application than bacterial strategies. Basidiomycota, representing the oligotrophic fungal group, decreased significantly under B7.5, whereas Ascomycota, representing the copiotrophic fungal group, increased significantly under the same treatment. Consequently, the fungal oligotroph/copiotroph ratio was lowest in B7.5. This result suggests that low-rate bio-compost shifted fungal communities toward a more copiotrophic strategy. Ascomycota often respond rapidly to easily available organic substrates, whereas Basidiomycota are commonly associated with the degradation of complex and more recalcitrant organic materials (Kurakov and Bilanenko, 2023). From an environmental perspective, this shift implies a faster turnover of the labile carbon pool, which may accelerate short-term nutrient mineralization; however, it also raises concerns about the reduced stability of soil organic carbon under such management regimes. The increase in Ascomycota under B7.5 may therefore reflect improved availability of labile carbon and nutrients derived from bio-compost, while the reduction in Basidiomycota indicates a relative weakening of oligotrophic fungal dominance. Interestingly, high-rate compost treatments did not produce the lowest fungal oligotroph/copiotroph ratio, suggesting that fungal life-history responses were not simply controlled by input amount. Instead, they may depend on interactions among substrate quality, nutrient stoichiometry, soil pH, and enzyme activity. This finding highlights that fungal communities may be more sensitive than bacterial communities to compost-induced changes in resource conditions and that moderate bio-compost input may be particularly important in shifting fungal ecological strategies.

Co-occurrence network analysis showed that compost application increased microbial network complexity, especially under B15. The B15 treatment had the largest numbers of nodes and edges in both bacterial and fungal networks, indicating more complex potential microbial associations. Organic amendments can provide diverse carbon compounds, nutrients, and microhabitats, which may support more taxa and increase opportunities for microbial co-occurrence (Wu et al., 2022; Cui J. W. et al., 2023). The particularly complex networks under B15 suggest that high-rate bio-compost may enhance microbial connectivity by combining high organic input with microbial inoculation effects. In the context of agroecosystems, a more complex and interconnected microbial network suggests greater functional redundancy and ecosystem resilience (Loreau et al., 2001), which are vital for buffering against environmental disturbances and maintaining stable biogeochemical cycling. Across all treatments, bacterial and fungal networks were dominated by positive correlations, indicating that co-occurrence patterns or shared ecological preferences were more common than negative associations. Positive links may reflect similar responses to compost-derived resources, cross-feeding relationships, or shared environmental niches, whereas negative links may indicate competition or niche differentiation (Yao et al., 2017; Luo et al., 2022). The low proportion of negative edges suggests that compost-amended soils mainly promoted coordinated microbial associations. However, CK had the highest proportion of negative bacterial edges, implying that resource limitation in unfertilized soil may intensify potential competition. The much simpler fungal network in CK also indicates that the absence of long-term organic input restricted fungal associations. Together, these results suggest that long-term compost application, particularly high-rate bio-compost, increased microbial network complexity and may enhance the ecological connectivity of soil microbial communities.

The relationships between microbial communities and environmental variables further indicate that soil phosphorus and oxidative enzymes were key drivers of microbial variation. Pearson correlation analysis showed that bacterial alpha diversity was mainly related to Cat and MnP activities, while fungal richness was positively associated with SOC, TN, TP, TN: TP, LiP, and Lac. Notably, bacterial Chao1 richness exhibited a significant negative correlation with soil moisture. This seemingly counterintuitive pattern might be explained by the fact that higher soil moisture can reduce soil aeration and oxygen supply by increasing water-filled pore spaces, thereby favoring moisture-tolerant or anaerobic taxa while limiting the ecological niches occupied by some aerobic bacteria (Gao et al., 2024). Therefore, this observed negative correlation may reflect the integrated response of bacterial communities to changes in the overall soil physicochemical environment, rather than an independent effect of soil moisture alone (Yang et al., 2022). Mantel tests also showed that bacterial and fungal community compositions were strongly correlated with TP, LiP, MnP, and Lac. These results suggest that microbial diversity and composition were closely linked to both nutrient availability and enzyme-mediated organic matter transformation. RDA further confirmed that MnP, LiP, and Lac were the strongest explanatory factors for microbial trophic group variation, with MnP showing the highest independent explanatory power. This emphasizes the importance of oxidative enzyme processes in shaping microbial life-history strategies, probably because oligotrophic and copiotrophic groups differ in their ability to utilize labile versus complex organic substrates (Min et al., 2024; Zhuang et al., 2024). The close association of Acidobacteriota, Ascomycota, and Bacteroidota with TP and oxidative enzymes suggests that these groups were favored by active nutrient cycling and organic matter transformation. In contrast, Basidiomycota, Alphaproteobacteria, and Chloroflexi were more closely related to pH and C: N: P stoichiometric ratios, indicating that their distributions were more strongly linked to background soil chemical conditions. Overall, this study demonstrates that long-term compost application reshaped microbial community structure, network complexity, and trophic strategies through changes in soil nutrient status and extracellular enzyme activities, with phosphorus availability and oxidative enzyme activity acting as central environmental drivers. Our findings highlight that optimizing bio-compost application, especially at higher rates, represents a promising agricultural strategy for enhancing soil fertility, promoting beneficial microbial processes, and potentially strengthening the resilience of agroecosystems to climate-induced stress.

We acknowledge several limitations in this study and propose corresponding future directions. First, the inference of microbial life-history traits from high-throughput sequencing data carries inherent uncertainties, and the co-occurrence networks constructed from a limited number of field replicates (n = 4) may not fully capture the ecological complexity of microbial interactions. Future studies employing metagenomic approaches, such as the Y-A-S framework, could provide more robust functional insights into life-history strategies and identify functional genes associated with fertility enhancement. Second, due to the specific scope of our study focusing on long-term stabilized effects rather than seasonal dynamics, we did not collect soil samples during the maize growing season. Future research incorporating seasonal comparisons would help elucidate the temporal dynamics of microbial responses to compost application. Third, the lack of comprehensive elemental analysis of soil samples limits our ability to explore the mechanistic regulation of oxidative enzyme activities by mineral elements. Future work integrating multi-element profiling would be highly valuable in this regard. Fourth, crop grain yields were not systematically recorded in this experiment. While the present study elucidates the mechanistic linkages among compost-derived nutrient inputs, oxidative enzyme activities, and microbial life-history strategies, future studies should integrate agronomic indicators, such as crop yield, to bridge the gap between microbial benefits and actual agricultural productivity.

## 5 Conclusion

This 30-year field study demonstrated that long-term compost application, particularly high-rate bio-compost, effectively enhanced soil fertility and reshaped microbial communities in a winter wheat–summer maize cropping system. The improvements in soil nutrient status and oxidative enzyme activities, coupled with the increased complexity of microbial networks and the shift toward copiotrophic fungal groups under moderate bio-compost input, underscore the potential of bio-compost to sustain soil functioning and ecosystem resilience. From an environmental perspective, these findings highlight that optimizing bio-compost application represents a promising strategy for enhancing soil carbon and nutrient cycling, thereby promoting a more complex and interconnected microbial network and supporting greater functional redundancy in agroecosystems.

## Data Availability

The datasets presented in this study can be found in online repositories. The names of the repository/repositories and accession number(s) can be found here: https://www.ncbi.nlm.nih.gov/bioproject/PRJNA1510276.
