# Supplementary material for: Effects of long-term application of traditional compost and bio-compost on soil microbial community structure and life-history strategies
Source: Front Microbiol. 2026 Sep 30;17:1910948. doi: 10.3389/fmicb.2026.1910948 (PMC13661530; doi:10.3389/fmicb.2026.1910948)
Supplement: Supplementary file 1 [file Supplementary_file_1.DOCX]

**Supplementary Information for**

**Effects of Long-Term Application of Traditional Compost and Bio-Compost on Soil Microbial Community Structure and Life-History Strategies**

Lizhan Liu^a,b†^, Tao Yang^b†^, Xiaofang Wu^a^, Yang Fan^c^, Peipei Zhang^b^, Jia Li^a,*^, Wenbing Tan^d^, Xiawei Peng^b, e, f,*^

^a^ Technical Centre for Soil, Agriculture and Rural Ecology and Environment, Ministry of Ecology and Environment, Beijing 100012, China

^b^ School of Biological Sciences and Technology, Beijing Forestry University, Beijing 100083, China

^c^ College of Resources and Environmental Sciences, China Agricultural University, Beijing 100193, China

^d^ State Environmental Protection Key Laboratory of Simulation and Control of Groundwater Pollution, Chinese Research Academy of Environmental Sciences, Beijing 100012, China

^e^ Institute of Tree Development and Genome Editing, Beijing Forestry University, Beijing 100083, China

^f^ Beijing Key Laboratory of Food Processing and Safety in Forestry, Beijing Forestry University, Beijing 100083, China

* Corresponding author.

E-mail address: lijia@tcare-mee.cn (J. Li); [xiaweipeng@163.com](mailto:xiaweipeng@163.com) (X. Peng).

^†^ These authors have contributed equally to this work

**Fig. S1.** Overview of the sampling area. (a) Geographical location of Hebei Province, China. (b) Location of Quzhou experimental station in Handan, China. (c) Distribution of long-term field experiment with different fertilization practices.

**Table S1.** Basic physicochemical properties of traditional compost and bio‑compost.

| Treatment | Traditional compost | Bio-compost |
| --- | --- | --- |
| OM (g/kg) | 358±25 | 444±34* |
| TN (g/kg) | 25±1.4 | 29±2.6 |
| AP (g/kg) | 5.2±0.9 | 20±1.6** |
| AK(g/kg) | 15±4.9 | 19±5.6 |
| pH | 7.4±0.3 | 7.7±0.4 |
| Moisture (%) | 40±1.3 | 37±1.7 |

**Note:** Values are means±standard deviations (n=3). Asterisks indicate significant differences between traditional compost and bio‑compost based on independent-samples t-tests (**P* < 0.05; ***P* < 0.01). OM, organic matter; TN, total nitrogen; AP, available phosphorus; AK, available potassium.

**Table S2.** Fertilization treatments of the field experimental soil

| Treatment | Fertilization condition | Application rate (t/ha) |
| --- | --- | --- |
| B15 | Bio-Compost | 7.5 |
| T15 | Traditional Compost | 15 |
| B7.5 | Bio-Compost | 7.5 |
| T7.5 | Traditional Compost | 15 |
| CK | Unfertilized control | 0 |
